# The extreme rainfall gradient of the Cape Horn Biosphere Reserve and its impact on forest bird richness

**DOI:** 10.1007/s10531-022-02353-5

**Published:** 2022-01-20

**Authors:** Claudio S. Quilodrán, Erik M. Sandvig, Francisco Aguirre, Juan Rivero de Aguilar, Omar Barroso, Rodrigo A. Vásquez, Ricardo Rozzi

**Affiliations:** 1 Department of Biology and Biochemistry, University of Fribourg, Fribourg, Switzerland; 2 Department of Zoology, University of Oxford, OX 1 3PS Oxford, United Kingdom; 3 Cape Horn International Center, Parque Etnobotánico Omora, Universidad de Magallanes, Puerto Williams, Chile; 4 Instituto de Ecología y Biodiversidad (IEB), Departamento de Ciencias Ecológicas, Facultad de Ciencias, Universidad de Chile, Santiago, Chile; 5 Centro de Investigación Gaia Antártica (CIGA), Universidad de Magallanes, Punta Arenas, Chile; 6 Sub-Antarctic Biocultural Conservation Program, Department of Philosophy and Religion & Department of Biological Sciences, University of North Texas, Denton, TX USA

**Keywords:** Forest birds, Hygric niche, Sub-antarctic ecoregion, Species richness, Precipitation gradient, Climate change

## Abstract

A natural laboratory is a place supporting the conditions for hypothesis testing under non-anthropogenic settings. Located at the southern end of the Magellanic sub-Antarctic ecoregion in southwestern South America, the Cape Horn Biosphere Reserve (CHBR) has one of the most extreme rainfall gradients in the world. Subject to oceanic climate conditions, it is also characterized by moderate thermal fluctuations throughout the year. This makes it a unique natural laboratory for studying the effects of extreme rainfall variations on forest bird communities. Here, we monitor the bird species richness in the different forest types present in the CHBR. We found that species richness decreased with increasing precipitation, in which an increase of 100 mm in average annual precipitation showed about 1% decrease in species richness. Similar patterns were found among different forest types within the CHBR. These results provide a baseline to investigate the interactions between physical and biotic factors in a subpolar region that climatically contrasts with boreal forests, which is subject to continental climatic conditions. This research highlights the importance of ecological and ornithological long-term studies in the CHBR, which can contribute both to a higher resolution of the heterogeneity of climate changes in different regions of the world, and to orient conservation policies in the Magellanic sub-Antarctic ecoregion in the face of growing development pressures.

## Introduction

A natural laboratory is a place that is sustained by multiple social, cultural and institutional dimensions, but a key feature is holding a unique set of geographic and/or biophysical attributes (Aguilera and Larraín 2021; Rozzi et al. 2006; Rozzi et al. 2020). Located at the southern end of the Magellanic sub-Antarctic ecoregion, the Cape Horn Biosphere Reserve (CHBR) stands out for its location and remarkable biophysical attributes, including (1) the absence of a biogeographic replicate globally, (2) housing the world’s southernmost rainforests, (3) having the cleanest rainwater on the planet, and (4) providing a baseline where it is possible to conduct research on ecosystems under pre-industrial conditions (see Rozzi et al. 2012; Rozzi et al. 2007). In this article we focus on an outstanding attribute, which has not been sufficiently investigated so far, the extreme rainfall gradient. This gradient span west to east, ranging from > 5,000 mm to < 500 mm of rainfall per year (Aguirre et al. 2021). This represents a unique situation worldwide, where in a west-east distance of 500 km of fjords, channels and islands, from the Pacific Ocean towards the Atlantic Ocean, rainfall decreases by an order of magnitude (Rozzi et al. 2004; Tuhkanen et al. 1990).

**Fig. 1:**
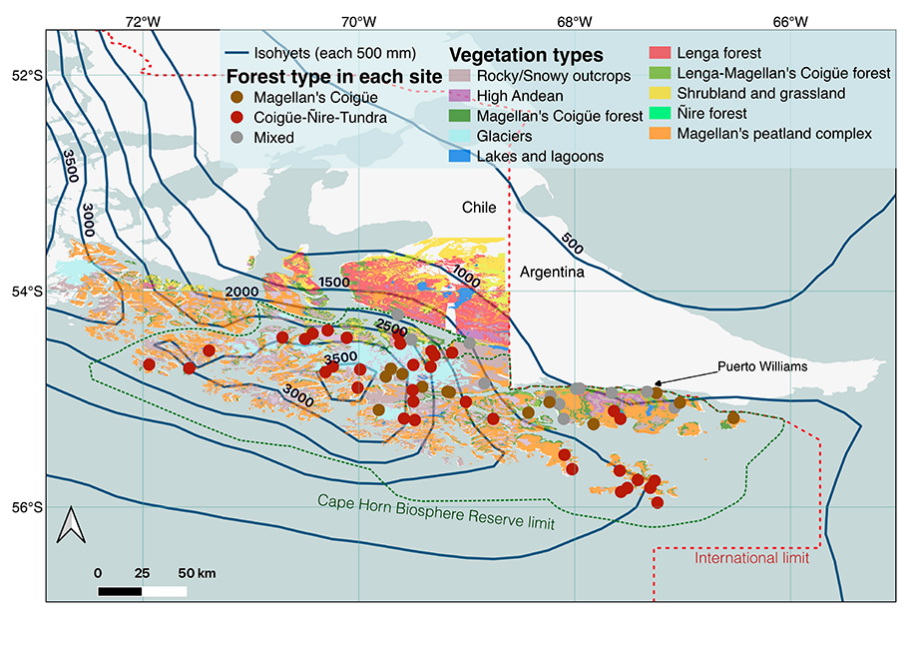
Rainfall gradient in the Cape Horn Biosphere Reserve. There are 61 sampling sites in the different type of forest that are represented with circles of different colors: mixed forest of coigüe and lenga (mixed, gray), coigüe-ñirre-tundra (CÑT, red), and Magellan’s coigüe (coigüe, yellow). The vegetation types are based on Rozzi et al. (2007). Solid lines and numbers represent precipitation isolines (mm). The precipitation information is extracted from the ERA5 database (see Methods)

The rainfall gradient of the CHBR is generated by the concurrent effects of the west wind circulation regime and the fragmented topographic distribution of the southern end of the Andes mountain range (Tuhkanen et al. 1990). This generates one of the most extreme precipitation gradients in the world (Aguirre et al. 2018; Carrasco et al. 2002; Garreaud et al. 2013), which in turn is associated with a marked variation in vegetation types within the CHBR (Rozzi et al. 2014) (Fig. 1).

The CHBR covers the southernmost area of the American continent. This area contains the southernmost forest ecosystems in the world (Rozzi et al. 2007) and, therefore, also contains the southern latitudinal limits of forest bird species. These forests offer an ideal natural laboratory given that the CHBR protects a large extension of the Magellanic sub-Antarctic ecoregion, which has been identified as one of the world’s remaining 24 pristine areas (“wilderness areas”) (Mittermeier et al. 2003). Despite representing a priority area for the conservation of sub-Antarctic flora and fauna (Muñoz et al. 1996; Rozzi and Jiménez 2014), the CHBR remains poorly studied in terms of ecological mechanisms and interactions in comparison with research carried out in similar northern hemisphere ecosystems (Rozzi and Jiménez 2014). In contrast to boreal forests, sub-Antarctic forests are subjected to oceanic climatic conditions; however, there is still little information regarding the influence of climatic factors on the assemblage of sub-Antarctic forest birds (Reyes-Arriagada et al. 2015).

The sub-Antarctic ecoregion differs from similar latitudes in the northern hemisphere as its climate is modulated by a much higher proportion of oceanic surface with respect to land surface (Schneider et al. 2003). The oceanic surface represents 98% of the area between 40°S and 60°S, whereas this surface represents 46% in the same latitudinal band in the northern hemisphere (Rozzi et al. 2012). This oceanic influence translates into lower thermal fluctuations in areas of the sub-Antarctic ecoregion with the same altitudinal range (Rozzi and Jiménez 2014; Schneider et al. 2003). The high rainfall gradient and naturally controlled thermal conditions offer a natural laboratory to evaluate the effect of extreme rainfall variations in the assemblage of forest birds. Furthermore, ecosystems at high latitudes are experiencing some of the fastest rates of climate change on the planet (Stuecker et al. 2018). Monitoring the ecological responses of sub-polar terrestrial biota to climate change has concentrated on the sub-Arctic (Rozzi et al. 2012). Yet, a complete understanding of the consequences of climate change at global scale requires the analysis of climate systems and the responses of biodiversity in both hemispheres.

The remoteness and low human presence in the CHBR constitute a refuge for species threatened by anthropogenic pressures, such as the Magellanic Woodpecker (*Campephilus magellanicus*) (Arango et al. 2007). However, the area is still threatened by the impacts of global climate change (Contador et al. 2014; Ulloa et al. 2012). The increase in CO2 levels, changes in temperature, and the alteration of hydrological cycles may lead to changes to the integrity of the ecosystems of the sub-Antarctic ecoregion (Mansilla et al. 2012).

In this study, we compile forest bird records across the CHBR, and characterize their assemblages spatially among different types of forest vegetation in relation to the rainfall gradient. We aim to determine the influence of rainfall gradient on the richness of forest bird species. This information provides a baseline to investigate interactions between physical and biotic factors in future studies in the context of climate change. It emphasizes the importance of inter-institutional collaborations to implement long-term studies, offering a new basis for the biocultural approach that has been implemented in the CHBR. Long-term studies can contribute both to the conservation of ecosystems in light of changing environmental conditions, and to the protection of unique organisms, such as the forest birds of the Magellanic sub-Antarctic ecoregion.

## Methods

### Study area

The CHBR protects 50,000 km^2^ of which 19,172 km^2^ are terrestrial ecosystems (Rozzi et al. 2006). It is located at the southern end of the sub-Antarctic ecoregion (Rozzi et al. 2012), where the dominant habitats are represented by forests of coigüe (*Nothofagus betuloides*), lenga (*N. pumilio*), ñirre (*N. antartica*), mixed *Nothofagus* forests, Magellan tundra complex (*Sphagnum* spp.), high-Andean regions, rocky outcrops, glaciers and scrublands, the latter predominantly composed of *Berberis* sp., *Ribes magellanicum*, *Embothrium coccineum*, *Drimys winteri*, *Chiliotrichum diffusum* and *Gaultheria XXXucronate* (Pisano Valdés 1977; Rozzi and Jiménez 2014). The composition of the understory varies in association with the rainfall gradient (Pisano Valdés 1977; Rozzi et al. 2006). In the more humid areas (> 2,000 mm of annual rainfall) the understory is dominated by liverworts (e.g., *Gackstroemia magellanica*, *Schistochilla lamelata*) and lichens (e.g., *Pseudocyphellaria* spp.) (Rozzi et al. 2008). In areas of intermediate humidity (1,000–2,000 mm of annual rainfall), the understory is dominated by mosses, woody ferns (including *Blechnum magellanica* and *Gleichenia pinnatifolia*), and shrubs such as *Philesia magellanica* (Rozzi et al. 2006). In less humid areas (< 1,000 mm of annual rainfall), woody ferns and *P. magellanica* are absent; in contrast, the understory is dominated by bushes that produce abundant berries, such as *Berberis ilicifolia*, *B. buxifolia* and *Ribes magellanicum* (Molina et al. 2016). In general, the climate is characterized by being hyper-humid, with low year-round temperatures and a very marked rainfall gradient (Schneider et al. 2003).

### Forest types

This study focuses on the forest environments of the CHBR, where we distinguish three types of forest vegetation across our 61 sampling sites (Fig. 1):

- Coigüe: coigüe (*N. betuloides*) evergreen forest, frequently associated with *Drymis winteri* and *Maytenus magellanica* understory.
- Mixed: mixed forest (evergreen and deciduous), dominated by coigüe (*N. betuloides*) and lenga (*N. pumilio*).
- Coigüe-ñirre-tundra (CÑT): mixed forest (evergreen and deciduous), dominated by coigüe (*N. betuloides*) and ñirre (*N. antarctica*), which is a forest of lower height or ‘stunted’, with open areas of tundra with peat bogs (primarily composed of *Sphagnum spp*).

### Bird monitoring

Ornithological monitoring has been carried out in the Omora Ethnobotanical Park and other study sites on Navarino Island since January 2000. This has involved sampling with mist nets and bird censuses on transects and point counts (Rozzi and Jiménez 2014). Monitoring has included two visits per year to 61 sites in the CHBR, one during the austral summer (October–April) and the other during the austral winter (May–September), distinguishing the records of migratory and resident birds. With these data, lists of forest bird species were prepared for the 61 sites within the CHBR (see Fig. 1), considering five main areas: (1) Navarino Island (18 sites), including Picton and Button Islands, Mascart Island, in the area of the Murray Canal and sites in the Ponsonby Sound, on Hoste Island; (2) northwest arm of the Beagle Channel and neighboring areas (16 sites), including sites on both sides of the northwest arm of the Beagle Channel, as well as neighboring fjords (Yendegaia Sound, Pía Sound, Ventisquero Sound) and Chair and Diablo Islands; (3) Seno Almirantazgo (12 sites), including neighboring fjords (Seno Parry, Ainsworth Bay, Seno Brooke), the valleys (Parallel River) and Caleta María, at the eastern end of Seno Almirantazgo; (4) areas west of the CHBR (six sites), including neighboring London and Basket islands; (5) Cape Horn archipelago area (nine sites), including the islands of the Cape Horn archipelago, as well as two sites on the eastern coast of the Hardy Peninsula (Orange Bay, Lort Bay) (see Fig. 1 for spatial locations of each monitoring site).

### Climatic variables

The particular conditions of the topography in these latitudes cause marked differences in rainfall on both sides of the Andes mountain range. The western slopes are frequently hit by low pressure fronts generating a large amount of precipitation, which increases with altitude due to the upward movement of air masses induced by the topography (Carrasco et al. 2002). Precipitation decreases abruptly when it passes to the eastern slopes, due to the Föhn effect (Weidemann et al. 2013). The rainfall gradient in the region can vary by more than 5000 mm, within an 80 km horizontal distance, and more than 2000 mm in a 300 m elevational range (Weidemann et al. 2018).

A report from the Intergovernmental Group of Experts on Climate Change (IPCC) highlights the lack of precipitation measurements (rain gauges) at a global level, which making it difficult to analyze rainfall at temporal and regional scales (Hartmann et al. 2013). The lack of rain gauges is particularly critical in southern Patagonia, with almost no representation on the western side of the Andes mountain range (Garreaud et al. 2013). In this study, we used mean annual rainfall information (total precipitation) from the ERA5 climate re-analysis (successor to ERA-Interim), which uses a large amount of historical observational data at a global level (e.g., satellite data, meteorological stations), modelling climatic variables with a spatial resolution of 0.25° (Copernicus Climate Change Service 2017). This database is frequently used to study the climate in remote environments with poor availability of meteorological stations (e.g., Sato and Inoue 2018; Wang et al. 2019). Average annual precipitation was estimated within the CHBR, using annual total precipitation data (mm) from the last 20 years (2000–2019, Fig. 1). In addition, we estimated the mean annual temperature (°C) from the ERA5 climate re-analysis by considering the monthly average across the same period of years. All precipitation and temperature data were extracted in an hourly format from the Copernicus Climate Data Store (CDS 2021). They were processed using Climate Data Operator (CDO) software (Schulzweida 2019) and GDAL (Open Source Geospatial Foundation 2021).

### Statistical analysis

The response variable represents the species richness (total number of species) observed at each of the 61 bird monitoring sites. This variable was log transformed to maintain the gaussian distribution of model residuals. The explanatory variables were the average annual precipitation (mm), average annual temperature (°C), and the type of forest. The variance inflator factor (VIFs < 1.5) indicated that there was no collinearity among explanatory variables. We included a spatial correlation structure between the 61 monitoring sites. Models were selected using the lowest value of the Akaike Information Criterion corrected for the sample size (AICc) (Burnham and Anderson 2002). The final model was a generalized least squares model (GLS) that considered a linear spatial correlation and a power variance structure (Zuur et al. 2009). Because the number of sites corresponding to the three types of vegetation has an unbalanced design, an ANCOVA with sum of squares of type III was considered. A Tukey test was used for multiple comparisons. All analyzes were carried out with the *R* program (R Development Core Team 2019). We used the *nlme* package (Pinheiro et al. 2019) for the GLS regression analyzes and the *emmeans* package (Lenth and Lenth 2018) for the multiple comparisons.

## Results

### Forest type

**Fig. 2:**
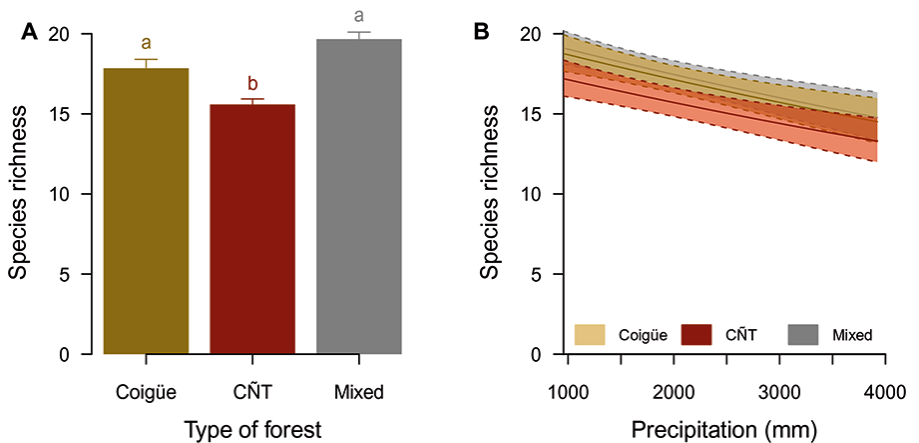
Effects of rainfall and forest type on the richness of forest birds. (A) Average species richness with their standard error. Different letters denote significant differences. (B) Impact of rainfall on species richness. The solid lines represent the estimated value in each type of forest. The dotted lines represent the standard error. The colors in both figures represent the type of forest: mixed forest of coigüe and lenga (gray), coigüe-ñirre-tundra (CÑT, red), and coigüe (yellow)

There is a homogeneous assemblage of birds, where most of the species detected are present in all types of forest (Table 1). Exceptions were the Blue-and-white Swallow (*Pygochelidon cyanoleuca patagonica*), which was not observed in the CÑT forests, and the House Sparrow (*Passer domesticus*), which was observed only in the coigüe forest, at a single monitoring site near the city of Puerto Williams. The Austral Parakeet (*Enicognathus ferrugineus*) and the Magellanic Woodpecker (*Campephilus magellanicus*) are found mainly in the mixed forests of coigüe (*N. betuloides*) and lenga (*N. pumilio*), and less frequently in the other two types of vegetation. More generalist species were found in 100% of the monitoring sites, such as the Thorn-tailed Rayadito (*Aphrastura spinicauda*), Patagonian Sierra-Finch (*Phrygilus patagonicus*), Rufous-collared Sparrow (*Zonotrichia capensis chilensis*), White-crested Elaenia (*Elaenia albiceps chilensis*), Dark-bellied (*Cinclodes patagonicus*) and Buff-winged Cinclodes (*Cinclodes fuscus*), Black-chinned Siskin (*Spinus barbatus*), and Austral Thrush (*Turdus falcklandii magellanicus*). The type of forest had a significant effect in explaining the richness of forest birds (F = 5.5, *p* = 0.006). The mean richness in coigüe forest was 17.9 ± 0.5 (mean ± standard error), in the mixed forest it was 19.7 ± 0.4 and in CÑT forest it was 15.6 ± 0.3. The first two forests were not significantly different (*t* = − 0.6, *p* = 0.81), but the CÑT forest presented significantly less species richness than the mixed (*t* = − 3.1, *p* = 0.008) and the coigüe (*t* = 2.8, *p* = 0.02) forests (Fig. 2A).

**Table 1:** Species observed in the different types of forest. The numbers represent the percentage of sites within each forest type with presence of the species: coigüe forest (12 sites), mixed forest of coigüe and lenga (13 sites), and forest of coigüe-ñirre-tundra (CÑT, 36 sites). Column S denotes migratory status of the species within the reserve. Resident species (R) were detected in both winter and summer surveys. Migratory species (M) were only detected in the summer monitoring

Family	Common name	Scientific name	S	Coigüe	Mixed	CÑT
Trochilidae	Green-backed Firecrown	*Sephanoides sephaniodes*	M	58	46	14
Strigidae	Austral Pygmy-Owl	*Glaucidium nana*	R	100	92	83
Picidae	Magellanic Woodpecker	*Campephilus magellanicus*	R	83	46	11
Psittacidae	Austral Parakeet	*Enicognathus ferrugineus*	R	83	31	14
Rhinocryptidae	Magellanic Tapaculo	*Scytalopus magellanicus*	R	100	100	100
Furnariidae	White-throated Treerunner	*Pygarrhichas albogularis*	R	100	100	97
Furnariidae	Buff-winged Cinclodes	*Cinclodes fuscus*	R	100	100	100
Furnariidae	Dark-bellied Cinclodes	*Cinclodes patagonicus*	R	100	100	100
Furnariidae	Thorn-tailed Rayadito	*Aphrastura spinicauda*	R	100	100	100
Tyrannidae	Tufted Tit-Tyrant	*Anairetes parulus*	R	100	100	89
Tyrannidae	White-crested Elaenia	*Elaenia albiceps*	M	100	100	100
Tyrannidae	Fire-eyed Diucón	*Xolmis pyrope*	R	100	100	89
Tyrannidae	Patagonian Tyrant	*Colorhamphus parvirostris*	R	92	69	14
Hirundinidae	Blue-and-white Swallow	*Pygochelidon cyanoleuca*	M	42	8	0
Hirundinidae	Chilean Swallow	*Tachycineta meyeni*	M	100	100	94
Troglodytidae	House Wren	*Troglodytes aedon*	R	100	100	94
Turdidae	Austral Thrush	*Turdus falklandii*	R	100	100	100
Fringillidae	Black-chinned Siskin	*Spinus barbata*	R	100	100	100
Passerellidae	Rufous-collared Sparrow	*Zonotrichia capensis*	M	100	100	100
Icteridae	Austral Blackbird	*Curaeus curaeus*	R	100	92	58
Thraupidae	Patagonian Sierra-Finch	*Phrygilus patagonicus*	R	100	100	100
Passeridae	House Sparrow	*Passer domesticus*	R	8	0	0

### Climatic variables

Rainfall had a significant effect on the three types of forest vegetation (*F* = 4.6, *P* = 0.03). Average annual rainfall in the mixed forest was 1371 ± 143 mm, in the coigüe forest it was 2172 ± 283 mm and in the CÑT forest it was 2400 ± 136 mm. The difference between the last two forests was not significant (*t* = − 0.8, *P* = 0.67), but the difference was significant between the mixed forest and the coigüe forest (*t* = 2.4, *P* = 0.04), as well as between the mixed forest and the CÑT forest (*t* = 3.8, *P* = 0.001). Rainfall had a negative effect on the richness of forest birds (Table 2). Richness decreased by about six species between the extreme values ​​of the precipitation range (~1000–4000 mm, Fig. 2B). Considering the coefficient of the linear model, each additional 100 mm of precipitation decreased the richness of forest birds by 0.9% (e^−8.7E−05·100^− 1, Table 2). Similar patterns of decreases in species richness with increasing precipitation occurred in the three forest types (Fig. 2B).

**Table 2:** GLS model on the richness of forest birds in the Cape Horn Biosphere Reserve. Species richness was log transformed. The model evaluates the effect of rainfall (mm), temperature (°C), and the type of forest

Variable	Coefficient	Standard Error	*t*	*p*-value
Intercept	3.02	0.13	23.25	< 0.01
Rainfall	− 8.7E-05	4.0E-05	− 2.15	0.03
Temperature	− 0.02	0.02	− 1.46	0.15
Forest:Mixed	0.11	0.03	3.11	< 0.01
Forest:Coigüe	0.09	0.03	2.77	< 0.01

Temperature was homogenously distributed among the three types of forests (*F* = 0.8, *P* = 0.41), with a mean annual temperature of 3.7 ± 0.2 °C. While temperature has no significant effect on forest bird species richness (*F* = 2.1, *P* = 0.15), a model that does not consider its effect was excluded, since the difference in AICc with the full additive model was greater than two units (ΔAICc = 2.7). Similarly, a model that considers the interaction between the type of forest and precipitation was also excluded because of the difference in AICc with the additive model (ΔAICc = 2.6). In this model, the interaction between explanatory variables was not significant (*F* = 1.8, *P* = 0.17), but a significant effect was observed for the precipitation (*F* = 6.3, *P* = 0.01) and the type of forest (*F* = 3.8, *P* = 0.03). This means that regression curves between precipitation and species richness, estimated for the three types of vegetation, differ in their intercept, but not in the slope of the curve (Fig. 2B). These results show the ubiquitous negative effect of precipitation on the bird assemblage in the three main types of forest of the Magellanic eco-region.

## Discussion

### Homogenous bird assemblage across forest types

The forest bird assemblage of the CHBR is relatively homogeneous across all major forest vegetation types. Most of the species are found in the three types of forests present in the reserve. However, variations in species richness are influenced by the rainfall gradient, where sites with higher average annual precipitation show lower species richness. The impact of the rainfall gradient is similar within and between vegetation types. While the mixed and coigüe forests differ in rainfall quantity, with similar species richness, they both show a pattern of species decline when increasing the amount of rainfall. In a similar way, the CÑT forest that does not differ in the rainfall amount from mixed forest, but differs in species richness also presents a similar trend of species decline with increasing rainfall. The impact of the rainfall gradient is therefore consistent across forest types of the Cape Horn Biosphere Reserve.

### Increasing precipitation in recent years

The intensity of the rainfall gradient in the region has increased in recent decades, influenced by a higher frequency of westerly winds (South Annular Mode or SAM) in a positive phase (Garreaud 2018). SAM contributes heavily (~35%) to climate variability in the Southern Hemisphere (Mayewski et al. 2009), and is characterized by positive (winds centered in Antarctica) or negative (winds centered between ~40–50 °S) tropospheric pressure anomalies (Moreno et al. 2014). During a positive phase, there is a compression in the western wind belt (westerlies), moving them further south, generating an increase in the wind intensities, which have a higher humidity load. Although precipitation increases on the western side of the Andes mountain, precipitation decreases in its eastern part, due to the Föhn effect (Moreno et al. 2010), resulting in an increase in the rainfall gradient. The highest frequency of SAM in the positive phase in recent decades, as well as in its projections for the near future, is related to global climate change (Clem et al. 2016; Garreaud 2018; Turner et al. 2016). In a similar way, climate change scenarios project an increase in the variability of annual rainfall and a higher frequency of extreme events (Coumou and Rahmstorf 2012). An increase in rainfall of 9% has been recorded in southern Patagonia during the La Niña phenomenon, in addition to an equivalent decrease during the El Niño phenomenon (Weidemann et al. 2018). The response of organisms to extreme climatic events is difficult to predict, but there is already evidence that it can lead to drastic changes at the ecosystem level and the collapse of populations (Harris et al. 2018). Our results provide the first evidence of the impact of the rainfall gradient on the most diverse group of vertebrates in the CHBR (Rozzi et al. 2006), and emphasize the importance of long-term monitoring of bird assemblages to respond according to the effects of abrupt changes in rainfall.

Abrupt changes in the forest ecosystem could particularly affect specialist species, such as the Magellanic Woodpecker and Austral Parakeet, which are more restricted to mixed forest of coigüe and lenga (Sandvig et al. 2020). In contrast, generalist species, such as Thorn-tailed Rayadito, observed in a wide latitudinal range, in a variety of forest habitats (Botero-Delgadillo et al. 2020; Moreno et al. 2007; Quilodrán et al. 2014), may be less affected. The abrupt climatic variations can also catalyze processes of habitat homogenization and favor the expansion of generalist habitat species (Frishkoff et al. 2016). This could be the case of the House Sparrow, a globally invasive and opportunistic species, but currently restricted to a single monitoring site in the CHBR, in the proximity of the town of Puerto Williams.

### The impact of increasing precipitation on the hygric niche

Rainfall is considered a key factor in the distribution of forest birds (Echeverri et al. 2019; Lindenmayer et al. 2019). In arid zones and tropical regions, the impact of rainfall has shown a trend contrary to that observed in this study, where richness increases with increasing rainfall (Echeverri et al. 2019; Seymour et al. 2015). In these areas, the productivity of the system and the resources available to forest birds increase with rainfall (Lindenmayer et al. 2019). In contrast, in areas of high rainfall, such as the Cape Horn rainforests, very high levels of rainfall can limit the habitat use of birds (Santillan et al. 2018) and decrease chick survival (Sandvig et al. 2017; Schöll and Hille 2020). In places with extremely high rainfall, a decrease in structural complexity of forests could also influence a lower species richness (Rozzi et al. 2007).

The difference in the response of bird richness to increasing rainfall between arid and wetter regions is consistent with the concept of hygric niche (Boyle et al. 2020). The hygric niche describes the range of precipitation conditions under which species can persist, and predicts that species living in drier regions can respond positively to precipitation increase, while the opposite is expected for species living in wetter regions. Boyle et al. (2020) describe this concept at the individual, population, and community level (species richness), but they limited their analysis for tropical systems, where the effect of seasonal temperature is less important than in temperate regions. We propose to extend this concept to the temperate and subpolar rainforest of the sub-Antarctic ecoregion, where the range of precipitation is extreme and the seasonal temperature variation is largely reduced because of the strong oceanic influence.

### A natural laboratory to assess the impacts of climate change

The CBHR is a natural laboratory that is ideal for assessing and monitoring the consequences of global climate change. First, this is an area with minimal human impact compared to subpolar ecosystems in the northern hemisphere, with no other counterpart in the southern hemisphere (Rozzi et al. 2014). Second, there is a direct impact of the ozone depletion and increasing ultraviolet radiation in the area (e.g., Mansilla et al. 2012; Rozzi et al. 2007; Ulloa et al. 2012). Third, the extreme rainfall gradient highlighted in this study is one of the most extreme rainfall gradients on the planet (Aguirre et al. 2018). Fourth, the impact of this rainfall gradient could be elucidated more clearly thanks to relatively homogenous temperature regime throughout the year, governed by the oceanic climatic conditions (Rozzi et al. 2012). This may allow a clearer distinction between the effects of precipitation and temperature, being a unique condition for studies about climate change and the hygric niche on temperate rainforest species. Precipitation and temperature are both considered to be critical to understanding species distribution and fitness (e.g., Deblauwe et al. 2016; Lany et al. 2020). However, most ecological studies evaluating the impact of climate change have focused on temperature rather than on precipitation (Boyle et al. 2020). The relatively poor knowledge regarding the effect of precipitation has a major influence on our ability to interpret and predict the impact of human induced climatic variations on biodiversity. Long-term biological studies performed in the CHBR have the potential to contribute to this knowledge gap.

### Biocultural conservation for preserving one of the last pristine areas

The CHBR is one of the few remaining places considered as pristine (“wilderness areas”) (Mittermeier et al. 2003), a condition that has been maintained largely due to its remote location and the control exercised by the Chilean Navy, which has administered the area as a military reserve (Rozzi et al. 2006). Increasing connectivity and tourist activity can represent a threat, if not regulated properly, as they may favor processes of homogenization of the landscape (Rozzi et al. 2007), including the introduction of invasive species (Schüttler et al. 2019). Additionally, global climate change poses a serious threat to the Magellanic sub-Antarctic ecoregion, potentially catalyzing modification in the characteristics of the habitat, migration patterns, as well as the distribution and reproductive phenology of numerous species (Coumou and Rahmstorf 2012).

We consider that the impact of the extreme rainfall gradient on forest birds has a biocultural dimension for two reasons. Firstly, because implementing long term studies on the climate and bird communities of this natural laboratory requires strong, close collaborations with the Chilean Navy, the National Forestry Corporation, the General Water Directorate and the Ministry of National Assets (Rozzi et al. 2006). In climate and biodiversity monitoring, these institutions offer both logistical support and the participation of technically trained personnel. Long-term monitoring is important to provide basic scientific information, essential for conservation policies and sustainable management of sub-Antarctic ecosystems and their avifauna (Barroso et al. 2020). Secondly, because changes in climate and bird life have cultural implications. One of the species who’s presence was most sensitive to the rainfall gradient is the Magellanic Woodpecker, which was an emblematic species in the creation of the CHBR (Arango et al. 2007). This species holds a special meaning in Yagan culture, whose cosmogony believes that in ancient times they were humans (Rozzi 2004; Rozzi et al. 2017). Finally, the Magellanic Woodpecker, and other birds that are sensitive to rainfall gradients and climate change, have the potential to attract birdwatchers. Responsibly implemented scientific tourism offers an economic opportunity for the CHBR, as well as a way to catalyze cultural transformations that favor the conservation of biocultural heritage.

## Data Availability

The dataset used in the analysis is publicly available on zenodo: 10.5281/zenodo.5856349.
